# Characterization of black crusts developed on historic stones with diverse mineralogy under different air quality environments

**DOI:** 10.1007/s11356-021-15514-w

**Published:** 2021-07-24

**Authors:** José Santiago Pozo-Antonio, Carolina Cardell, Valeria Comite, Paola Fermo

**Affiliations:** 1 CINTECX, GESSMin group, Dpto. de Enxeñaría de Recursos Naturais e Medio Ambiente, Universidade de Enxeñaría de Minas e Enerxía, Universidade de Vigo, 36310 Vigo, Spain; 2 Department of Mineralogy and Petrology, Faculty of Science, University of Granada, 18071 Granada, Spain; 3 Dipartimento di Chimica, Via Golgi 19, Università degli Studi di Milano, 20133 Milan, Italy

**Keywords:** Black crust, Gypsum, Architectural heritage, Atmospheric pollution, Stone alteration, Preventive conservation

## Abstract

Black crusts (BCs) are one of the most critical alteration forms found on stones belonging to architectural heritage. Since they could be considered as passive samplers of atmospheric pollution, it would be plausible to establish relations between the air contamination and the BCs. With this aim, we have characterized BCs collected on historic buildings from two Spanish cities (Granada and Vigo) with different polluted atmospheres, as well as formed on stone substrates of varied mineralogy and texture. Likewise, in order to assess the impact of the atmospheric pollutants on the growth of BCs, quartz fiber filters were used as surrogate substrates and placed nearby the studied buildings to collect and analyze the aerosol particulate matter (PM). To this end, an array of complementary analytical techniques was used to evaluate the mineralogy, chemical composition, and texture of the BCs and to establish the correlation with the ions, OC (organic carbon), and EC (elemental carbon) detected in the PM on the quartz fiber filters. As result, BCs developed on carbonate substrates from Granada show more complex structure than those from Vigo, which are thinner because of frequent rain episodes. In both cities, NaCl, Pb-Cl, and Ca-Cl-rich particles, Ca-phosphate particles and clusters of Ba-sulfate-rich particles were detected. However, metal-rich rounded particles were more abundant in Granada’s BCs, including soot particles. BCs from Granada were richer in carbonaceous components (OC and EC) than the Vigo’s BCs. Although in the filters PM did not show EC—mainly due to traffic—, in the BCs from both locations OC and EC were detected. Therefore, this different composition was related to the mineralogy of the stones and the higher pollution of Granada in contrast to the industrial and sea-exposed city of Vigo.

**Supplementary Information:**

The online version contains supplementary material available at 10.1007/s11356-021-15514-w.

## Introduction

Black crusts (BCs) are one of the most dangerous alteration forms of stones in architectural heritage worldwide, regardless of the mineralogy and texture of the stone substrate (ICOMOS 2008; Pozo-Antonio et al. 2017). Indeed, BCs compromise the durability and esthetic appearance of the historic buildings and monuments (Rivas et al. 2014; Comite et al. 2020). They appeared firmly adhered to the substrate on area protected against direct rainfall or water runoff in urban environment (ICOMOS 2008). BCs are composed by a matrix primarily made of gypsum (CaSO_4_.2H_2_O) and/or other calcium sulfates such as anhydrite (CaSO_4_) or bassanite (CaSO_4_.1/2H_2_O). (ICOMOS 2008; Pozo-Antonio et al. 2017). In general terms, calcium sulfates occur easily on carbonate stones (marble, limestone, travertine, etc.) due to the interaction of the acidic component SO_4_^2−^ primarily from air pollution through dry deposition, or dissolved in water with Ca^2+^ which is released by dissolution processes from the calcite of the substrate (El-Gohary 2010; Ruffolo et al. 2015; Comite et al. 2020). Sulfur can come also from deposition of sea spray in cities with strong maritime influence (Rivas et al. 2014) and from the sulfate-rich cements (Rivas et al. 2014; Morillas et al. 2016a). During BCs growth, gypsum crystals trap C-particles giving to the crusts their typical black color. As well metal particles are embedded in BCs (Gulotta et al. 2013; Rivas et al. 2014; Fermo et al. 2015; La Russa et al. 2017; Pozo-Antonio et al. 2017; Silva et al. 2020; Comite et al. 2020), mainly Pb, Fe, and Zn-rich particles from lead gasoline (Rodriguez-Navarro and Sebastian 1996; Geller et al. 2006; Comite et al. 2020) employed until about 25 years ago. Likewise, Cu, Ni, Cr, and V are trapped in the BCs that come from the use of other combustibles such as oil, diesel, and gasoline (Rodriguez-Navarro and Sebastian 1996; Dongarrà et al. 2009; Comite et al. 2020), which were widespread after the abolition of leaded gasoline.

In silicate stones, such as granite, despite their low or absent calcium content, thick and compact gypsum-rich BCs have been also found (Sanjurjo Sánchez et al. 2009, 2011; Rivas et al. 2014). Ca^2+^ may come from hydrolysis processes of plagioclase mineral grains under acid environments (Simão et al. 2006; Pozo-Antonio et al. 2017). In other investigations, it was found that Ca^2+^ comes from cement and mortar dissolution or old lime remains (Rivas et al. 2014).

As BCs have been considered as a passive sampler of atmospheric pollution (Ausset et al. 1999; Török 2008; Urosevic et al. 2012; Comite et al. 2020), compounds found in BCs can be used as an indicator of environmental pollution, and also of climate change (De Kock et al. 2017). Therefore, cities showing different pollution levels and scenarios will show BCs with dissimilar compositions and diverse structural complexity levels.

Comite et al. (2020) have characterized two different BCs groups from marble stones belonging to the façade of cathedral of Monza (Italy). On the one hand, BCs collected in the upper part of the building showing simple structures; on the other hand, more complex BCs placed closer to the bottom of the building, with higher amount of metal particles mainly Pb-rich particles mixed with Cl, due to the grater accumulation time.

Attending to carbonaceous species found in BCs, it could be considered that the total carbon (TC) is composed of carbonate carbon (CC), elemental carbon (EC), and organic carbon (OC). In carbonate stones, such as marbles or limestone, CC derives from the substrate; high CC values in BCs indicate the great alteration of carbonate stones underneath (Comite et al. 2020). EC has a primary origin emitted by combustion processes—traffic or biomass burning (Fuzzi et al. 2006; Gentner et al. 2012; Piazzalunga et al. 2013). OC is both of primary origin (Piazzalunga et al. 2013; Vassure et al. 2014; Daellenbach et al. 2016) and of secondary origin, because it is formed from gaseous precursors such as volatile organic compounds (Robinson et al. 2007; Bernardoni et al. 2011; Gentner et al. 2012).

The aim of this paper is to analyze the influence of the mineralogy, texture, and deterioration of historic stones as well as the pollution levels they are exposed on the composition and microstructure of BCs formed on architectural heritage surfaces. Then, BCs from historic buildings from two cities with distinct heritage stones and pollution scenarios, i.e., Vigo (NW Spain) and Granada (SE Spain) were collected. Consequently, in Vigo BCs were collected from an hercynian granite while in Granada, BCs were taken from calcarenite, travertine, and marble. The chemical and mineralogical composition and the texture and structure of the BCs were investigated. The analytical techniques used for the characterization of BCs were X-ray diffraction (XRD), Fourier transform infrared spectroscopy (FTIR), stereomicroscopy (SM), polarized light microscopy (PLM), and high-resolution scanning electron microscopy coupled with energy dispersive X-ray spectroscopy (HRSEM-EDX), thermogravimetric analysis (TGA) coupled with carbon, hydrogen, and nitrogen (CHN) analysis, and ion chromatography (IC). Moreover, thermal-optical transmittance (TOT) and ion chromatography (IC) were applied to analyze the aerosol particulate matter (PM) collected with filter placed nearby the BCs.

## Materials and methods

### Sampling: BCs and quartz fiber filters

**Fig. 1:**
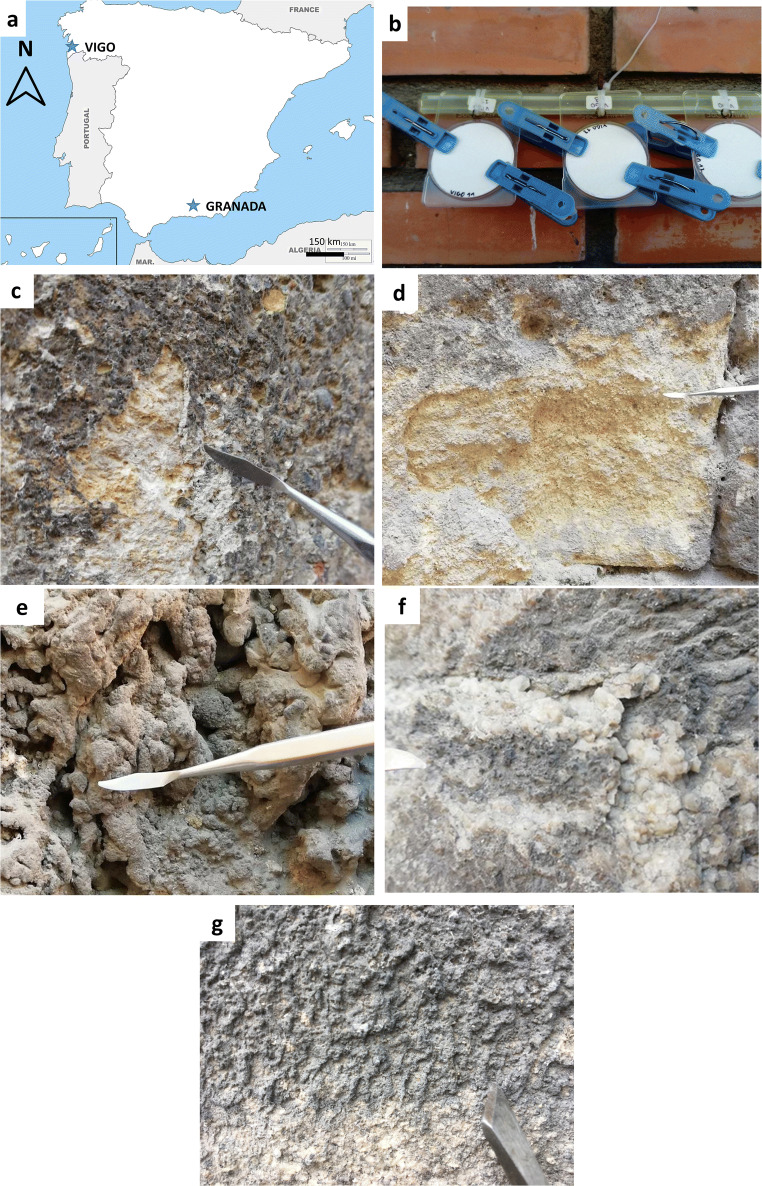
**a** Vigo and Granada location in Spain. **b** Quartz filters (VI1-3) placed in the center of Vigo during 10 months. **c**–**f** Macroscale view of BCs from Granada historic buildings. **c** BC on calcarenite at the Monastery of *San Jerónimo* (SJ-C-GR), **d** BC on calcarenite at the cathedral (CAT-C-GR). **e** BC on travertine at the Church of *San Justo y Pastor* (SJP-T-GR). **f** BC on marble at the *Hospital of San Juan de Dios* (SJD-MA-GR*.* **g** Macroscale view of BCs from Vigo buildings. BC on granite at Asylum *Ancianos Desamparados* (AS-G-VI)

BCs were taken from the bottom (up to 2-m height) of historic buildings located in different air pollution scenarios in Granada (5 BCs) and Vigo (6 BCs) (Table 1, Fig. 1a, c–f). Small samples were extracted with a hammer and a chisel to ensure that stone underneath the BC was also obtained in order to study the crust-substrate boundary. In Granada, the visual inspection of the famous Alhambra monument (Unesco World Heritage site 1984) located on top of the Sabika hill at ca. 150 m above the city center (ca. 700m a.s.l.) and surrounded by gardens and a forest, revealed the lack of BCs in its construction materials. BCs identification codes were based on the historic building they come from as follows (Table 1): CAT for Cathedral, CC *for Corral del Carbón*, SJ for Monastery of *San Jerónimo*, SJD for *Hospital of San Juan de Dios*, and SJP for the Church of *San Justo y Pastor*. Next appears the initial of the carbonate substrate: C for calcarenite (type of limestone), T for travertine, and MA for marble, and finally, the identifier for the city, i.e., GR for Granada.

**Table 1:** BCs sampled from historic buildings (up to 2- m height) at the city center of Granada and Vigo

City	Location	Orientation	Environment	Substrate	ID
Granada	Cathedral (CAT)	NW	In the past, it was facing a heavy traffic street	Calcarenite	CAT-C-GR
	*Corral del Carbón* (CC)	E	Close to a heavy traffic street	Travertine	CC-T-GR
	Monastery of *San Jerónimo* (SJ)	S	Close to a heavy traffic street	Calcarenite	SJ-C-GR
	Hospital of *San Juan de Dios* (SJD)	S	Facing a heavy traffic street	Marble	SJD-MA-GR
	Church of *San Justo y Pastor* (SJP)	NW	Facing a heavy traffic street	Travertine	SJP-T-GR
Vigo	Asylum *Ancianos Desamparados* from *Pi i Margal* street (AS)	SE	Heavy traffic street	Granite	AS-G-VI
	Asylum *Ancianos Desamparados* from *Santa Marta* street (SM)	W	Heavy traffic street	Granite	SM-G-VI
	Asylum *Ancianos Desamparado*s from *Angustias* street (AN)	S	Heavy traffic street	Granite	AN-G-VI
	Bonin building in a street close to the sea (A)	N	Heavy traffic street close to the sea	Granite	A-G-VI
	Building in the heavily trafficked *Elduayen* street (E)	NE	Heavy traffic street	Granite	E-G-VI
	Building in the heavily trafficked *Paseo de Alfonso* street (PA)	NW	Heavy traffic street	Granite	PA-G-VI

The building, orientation, environment, substrate, and ID are mentioned. *C* calcarenite, *T* travertine, *MA* marble, *G* granite, *GR* Granada, *VI* Vigo

At Vigo’s old quarter, BCs were sampled from four historic buildings (Table 1, Fig. 1a, g) constructed with leucogranite and two-mica granite, both common local stones (IGME 1985). As reported in Rivas et al., (2014), the joint mortar between granite ashlars was made of Portland cement mixed with fine-grained granitic aggregate; the ashlars were laid using sand and concrete. BCs were labeled considering either the name of the sampled building or the street where that construction is located (Table 1), as follows: AS, SM, and AN for the asylum *Ancianos Desamparados* (each BC was taken from different orientated walls of the building corresponding to different streets-Table 1), A for the *Bonin* building in the *Areal* street, E for a building in the heavily trafficked *Elduayen* street, and PA for a building in the also heavily trafficked *Paseo de Alfonso* street. Next, the letter G means the granite substrate underneath, and finally appears the identifier for the city, i.e., VI means Vigo.

Quartz fiber filters were exposed vertically (Fig. 1b) during 10 months in specific places at different heights and orientations near the sampled buildings in order to be exposed to similar polluted scenarios and collect atmospheric PM in a passive way (dry deposition) (Table 2). Filters were kept inside an open box to protect them from direct rain impact (Fig. 1b); they were weighed before and after environmental exposure. The filter identification code is formed by the acronym of the city followed by a number (GR for Granada’s filters and VI for Vigo’s filters, Table 2).

**Table 2:** Quartz fiber filters placed nearby the sampled buildings to collect the atmospheric particulate matter (PM) in Granada and Vigo

City	Filters	Location (environment)	Height (m)	BCs closed to these filters	ID
Granada	3 and 4	*Albaizín* quarter; first-floor balcony at S orientation facing a pedestrian street	4		GR3,4
	7	E orientation. Second-floor balcony in the city center (Faculty of Science) facing a heavy traffic avenue and exposed to rain events	7	SJP-T-GR	GR7
	8	NW orientation. First-floor balcony of a private house near the Monastery of *San Jerónimo*, in a pedestrian street. Well protected from rain and direct impact of traffic pollution	4	SJ-C-GR SJD-MA-GR	GR8
	11 and 12	NE orientation. First-floor balcony of a private house located in the city center at the confluence of 4 heavy traffic streets. Filters highly exposed to air pollution and rain impact	4	CAT-CC-GR CC-T-GR	GR11,12
Vigo	1, 2, and 3	SE orientation. Second-floor balcony of a private house located in the city center at the confluence of 2 heavy traffic streets. Well protected from rain	6	A-G-VI	VI1, 2, 3
	4 and 5	NW orientation. First-floor balcony of a private house facing a heavy traffic avenue. Well protected from rain	5	AS-G-VI E-G-VI SM-G-VI AN-G-VI PA-G	VI4,5
	6 and 7	NW orientation (6) and SE orientation (7). Second-floor balcony of a private house facing a heavy traffic avenue and orientated to the sea. Well protected from rain	6	AS-G-VI SM-G-VI AN-G-VI PA-G-VI	VI6,7

The number of the filter, location (environment), height, collected crusts nearby, and filter ID is mentioned. Check Table 1 to recognize BCs IDs. *C* calcarenite, *T* travertine, *MA* marble, *G* granite, *GR* Granada, *VI* Vigo

### Analytical techniques

Firstly, the BCs were studied using a stereomicroscope (SM), *model SMZ 1000* (Nikon, Japan) which incorporates a photomicrography system, to determine their structure and superficial characteristics. Next, small BCs scales were embedded in acrylic resins to obtain polished thin sections in order to study their cross sections by polarized light microscope (PLM) in transmitted and reflected light (*Carl Zeiss Jenapol U* instrument, Germany). The PLM was equipped with a digital camera (*Nikon D-7000*) which allowed us to unravel the mineralogy, texture, and structure of the BCs.

X-ray diffraction (XRD) was applied to identify and semi-quantify the mineralogy of the BCs using a *Siemens D5000*. To this end, ca. 0.1 mg of each BC was separated from the substrate with a scalpel. XRD analytical set up conditions were Cu-Kα radiation, Ni filter, 45-kV voltage, and 40-mA intensity. BCs samples were explored in the range between 3 and 60° 2θ with 0.05° 2θ s^−1^ goniometer speed. The *X’Pert HighScore* software (*Malvern Panalytical B.V*., The Netherlands) was used to identify the mineral phases.

The same powder BC sample was studied by Fourier transform infrared spectroscopy in attenuated total reflectance (ATR-FTIR) mode to ascertain its molecular composition using a *Thermo Nicolet 6700*. The infrared spectra were recorded from 400 to 4000 cm^−1^ at 2-cm^−1^ resolution over 100 scans.

Furthermore, the BCs bulk samples and the polished thin sections were analyzed via a high-resolution scanning electron microscopy (HRSEM) using a *Supra 40Vp Carl Zeiss* (Germany) to investigate their composition, microtexture, and microstructure. The microscope was equipped with secondary electrons (SE) and backscattered electron detectors (BSE) that provide topographical and chemical images, respectively, as well as a microanalysis system (*Aztec 3*) to deliver elemental information by means of energy dispersive X-ray spectroscopy (EDX). Both bulk and thin section samples were carbon-coated to be studied under high vacuum level. EDX single point analyses and X-ray maps were acquired from specific areas to better recognize the position, quantity, and morphology of crystalline/amorphous phases present in the BCs.

In order to identify the ions present in both the BCs and the PM collected in quartz filters, ion chromatography was applied using a *ICS-1000 HPLC* system equipped with a conductivity detector (Piazzalunga et al. 2013; Comite et al. 2020). Likewise, the determination of OC (organic carbon) and EC (elemental carbon) was carried out on both the BCs and the PM from the quartz fiber filters. The filters were analyzed using a thermal-optical transmittance (TOT) *Sunset* instrument following the methodology conventionally used for the PM (Piazzalunga et al. 2013), while BCs samples were analyzed by thermogravimetric analysis (TGA) coupled with carbon, hydrogen, and nitrogen analysis following a methodology reported and specifically set up for OC and EC analysis in BCs (Fermo et al. 2015; Comite et al. 2020).

## Results

### Environmental and air quality data of the sites

The city of Granada (SE Spain) has a Mediterranean climate with semi-continental influence. It is located in a geological depression at the foot of the Sierra Nevada mountains that reach ca. 3500-m elevation. The climate is characterized by significant diurnal T and RH variations of around 20 °C and 50% respectively (Herrera et al. 2018). Average maximum *T* is ~40 °C in summer, and the minimum *T* is ~−3 °C in winter, while average RH is ~40% in summer and ~75% in winter (Velilla n.d., accessed on January 2021). Figure S1 shows the average air contamination values of SO_2_ (Figure S1a), NO_2_ (Figure S1c), PM10 (particles with a diameter of 10 μm, Figure S1e), and PM2.5 (particles with a diameter of 2.5 μm, Figure S1g) of the last 10 years (2010–2019, Informes Ecologistas en Acción 2010–2019). In spite of being a non-industrialized city, accumulation of fine PM is important due to heavy traffic and intensive construction works (Horemans et al. 2011; Urosevic et al. 2012). Topography and low wind speeds in Granada facilitate this PM accumulation (Horemans et al. 2011; Urosevic et al. 2012). In fact, PM10 and PM2.5 (Figure S1e and g) exceeded the recommendations issued by the World Health Organization (WHO) in most cases, which are more restrictive than those established by the Directive 2008/50/EC. For SO_2_, it exceeded the limit recommended by the WHO in 2015. Indeed, Granada is among the cities with the highest SO_2_ contamination in Western Europe according to the European Environment Agency (Herrera et al. 2018).

The city of Vigo (NW Spain) which is one of the most important Atlantic port cities in terms of industrial, commercial, fishing, and shipbuilding activities, has a humid sub-tropical climate, with rainy winters (1800-mm rainfall) (Martínez-Cortizas 1987; Martínez-Cortizas and Pérez 1999) marked by low-pressure S-SW fronts from the Atlantic Ocean. Vigo shows an average temperature of 13 °C and a low seasonal thermal oscillation (average of the minimum temperature values of 8 °C and average of the maximum values of 19 °C). RH remains high, above 63% during the entire year. Considering SO_2_ (Figure S1b), NO_2_ (Figure S1d), PM10 (Figure S1f), and PM2.5 (Figure S1h) concentrations for the last 10 years (2010–2019, Informes Ecologistas en Acción 2010-2019), PM10 and PM2.5 in Vigo exceeded those recommended by the WHO in several occasions, but SO_2_ and NO_2_ levels were always below the limits established by the Directive 2008/50/EC and the WHO.

### BCs characterization

**Fig. 2:**
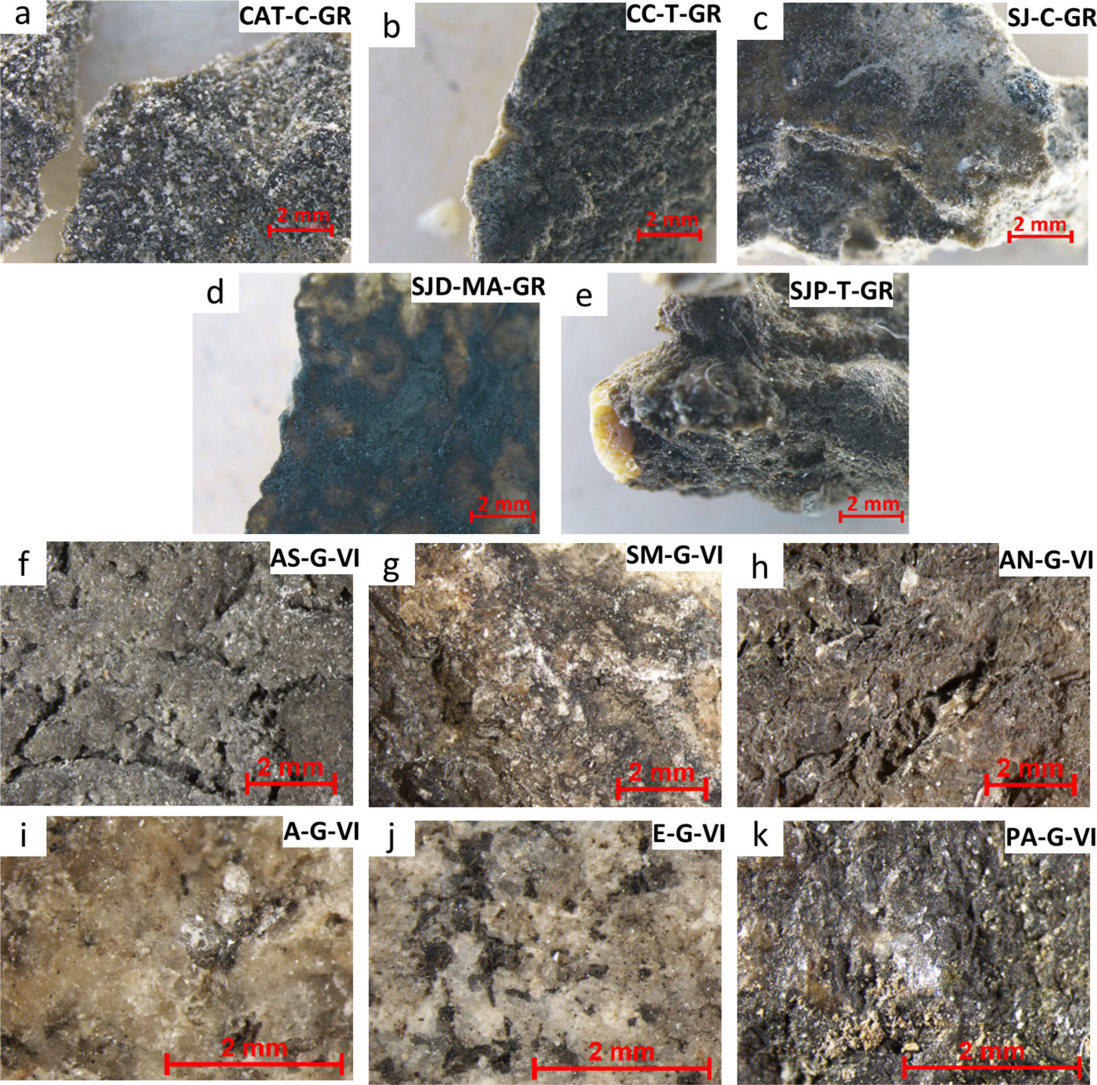
Stereomicroscopy micrographs (× 10) of the BCs from Granada (**a**–**e**) and Vigo (**f**–**k**) (Check Table 1 to recognize the BCs IDs)

BCs from Granada were firmly attached to the substrate (Fig. 2a–e) These BCs were quite homogeneous in appearance and continuity. Regarding Vigo’s BCs (Fig. 2f–k), the darkest ones were those collected in the ancient asylum (Fig. 2f–h) and PA-G-VI (Fig. 2k). Among them, AS-G-VI, which was the one located in the place most protected against rain and winds, showed the greatest thickness (Fig. 2f). In the E-G-VI crust (Fig. 2j), the rock-forming minerals were clearly distinguished.

**Fig. 3:**
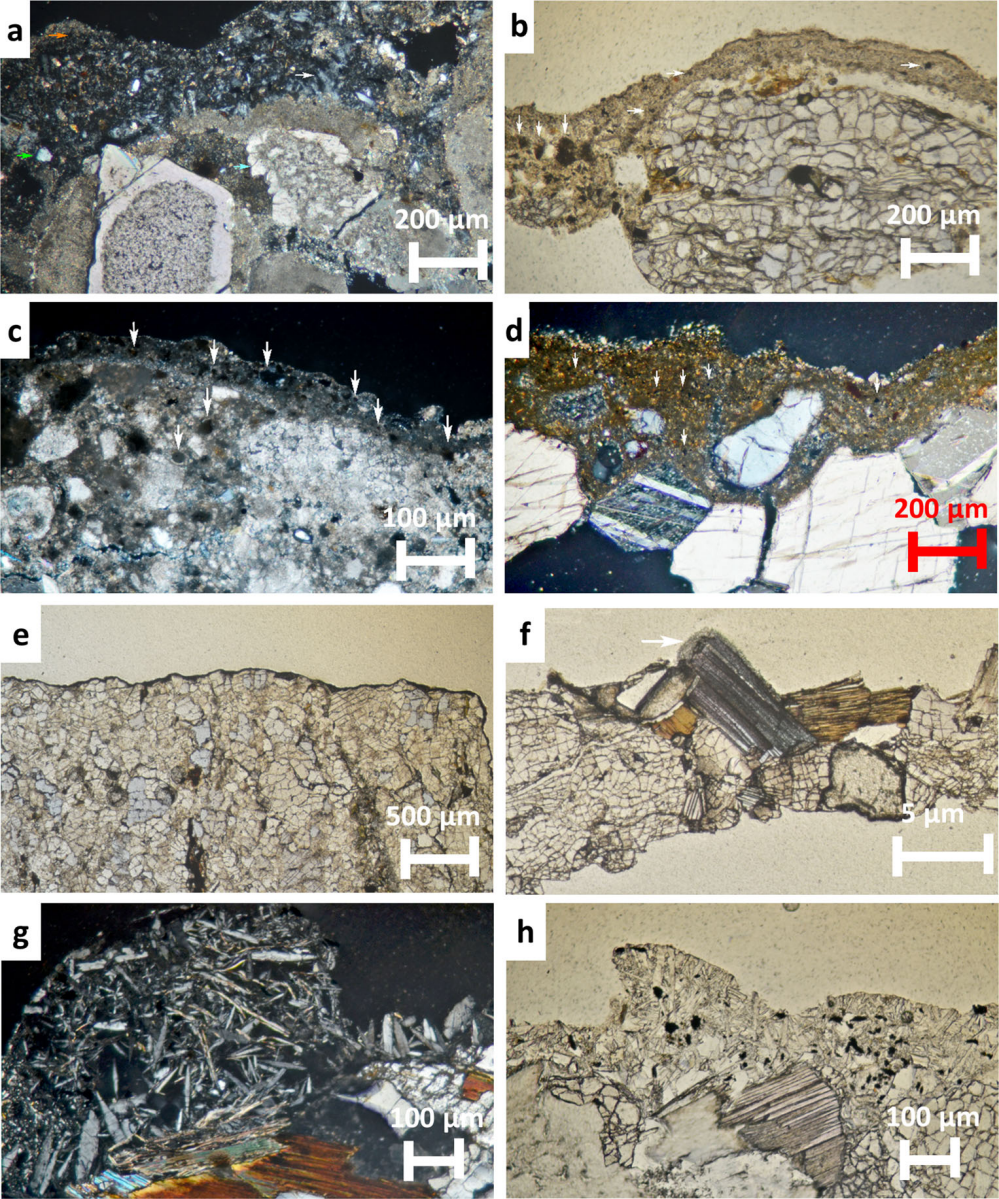
a–d PLM micrographs of BCs (thin sections) from Granada. a CAT-C-GR (crossed polars); note the gypsum crystals (white arrow), quartz grains (green arrow), clay minerals (orange arrow), and dog’s tooth calcite crystals (blue arrow). b SJ-C-GR (parallel polars); note the embedded soot particles (white arrow) and the intensely cracked calcite grains. c SJP-T-GR (crossed polars); note the abundant soot particles (white arrows). d SJD-MA-GR (crossed polars); note the embedded soot particles (white arrow). e–h PLM micrographs of black crusts (thin sections) from Vigo. e PA-G-VI showing typical thin and homogeneous layer (parallel polars). f A-G-VI with N orientation and exposed to the direct sea effect (parallel polars); note the massive tiny gypsum crystals above and among the feldspar crystals (white arrow). g AS-G-VI displaying a thick layer of arrowhead and lenticular gypsum crystals which also grow inside the mica and feldspars crystals (crossed polars). h AS-G-VI enclosing copious soot particles (parallel polars) (For interpretation of the references to color in this figure legend, the reader is referred to the web version of this article)

The PLM study of the Granada’s BCs revealed that all were largely composed of acicular crystals (better grown in the calcarenite crusts-CAT-C-GR, SJ-C-GR-Fig. 3a, b) in close association with black soot particles (which were far more abundant in Granada’s BCs than in Vigo’s BCs), quartz grains, and clay minerals. Crusts thickness on calcarenite-CAT-C-GR, SJ-C-GR- ranged from 180 to 360 μm (Fig. 3a, b), while on travertine-CC-T-GR, CJP-T-GR- ranged from 180 to 260 μm (Fig. 3c), and were below 200 μm in marble-SJD-MA-GR- (Fig. 3d). Carbonate substrates displayed different damage levels according to their nature. Thus, whereas the travertine displayed minor dissolution-precipitation patterns located precisely on the contact with the BC (Fig. 3c), the marble crystals showed fissures (Fig. 3d) that ultimately lead to granular disaggregation. Calcarenite was the most strongly weathered stone, suffering from intense granular disaggregation (Fig. 3b) and showing dissolution-precipitation features leading to the growing of dog’s tooth (secondary) calcite crystals (Fig. 3a).

The PLM analysis of Vigo’s BCs revealed that all granite substrates were severely damaged with the quartz crystals deeply fractured, and feldspar and mica crystals delaminated due to gypsum precipitation (Fig. 3e, f). The typical thickness for all crusts was less than 50 μm irrespective of their orientation (Fig. 3e, f). They were quite homogeneous and made of massive fine-grained gypsum crystals together with copious clay minerals and soot particles which contributed to their brownish and dark-grayish color respectively. The exception was the AS-G-VI crust (SE oriented) which was sampled from a granite well-protected to the environment (rain and wind) (Fig. 3j). This crust was made of a thick non-continuous layer made of a loosely cluster of arrowhead and lenticular gypsum crystals (up to 650 μm) which also grew among feldspars and mica crystals starting their delamination (Fig. 3g). PLM examination also showed that this gypsum matrix entrapped abundant soot particles (Fig. 3h).

Therefore, visual and PLM study of BCs from Granada and Vigo revealed that they differed mostly in thickness and color. BCs in Vigo were thinner and lighter that those from Granada (Fig. 3). These characteristics should be assigned to the different formation processes governed by the stone mineralogy, the different air pollution composition, and varied climate of both cities (Figure S1). In Granada, Ca^2+^ easily released by dissolution processes from the calcite of the carbonate substrates reacts with SO_4_^2−^ to ultimately form gypsum (El-Gohary 2010; Rivas et al. 2014; Ruffolo et al. 2015; Comite et al. 2020). In contrast, the quantity of available Ca^2+^ is much reduced in granite (Rivas et al. 2014). Recall that Granada showed higher levels of atmospheric pollutants than those measured in Vigo in the last 10 years (Figure S1). The dry climate conditions usually found in Granada together with the high levels of airborne dust particles and SO_2_ have assisted the formation of thick and dark BCs (Urosevic et al. 2012). On the other hand, it should be noted that all crusts from Vigo were sampled from granite buildings placed in streets with heavy traffic. Consequently, their dissimilar features in terms of structure, texture, and composition should be related to their orientation. On the contrary, Granada’s BCs were sampled from different carbonate stone substrates (calcarenite, travertine, and marble), the results revealing that the BCs features (color, substrate adherence, width, and microstructure) were different according to the substrate nature (Urosevic et al. 2011, 2012; Luque et al. 2011) and the air pollution scenario under they were growing, rather than on orientation.

Attending to the XRD results (Table 3), all the crusts from Granada were composed of gypsum except CC-T-GR that shows a black layer (Fig. 2b) composed essentially by (CaCO_3_) and dolomite (CaMg(CO_3_)_2_. Abundant calcite was also found in CAT-C-GR while quartz (SiO_2_) was only identified in SJD-MA-GR. In Vigo, mineral composition of BCs was more heterogeneous than that of Granada BCs because granite-forming minerals (quartz, albite-NaAlSi_3_O_8_, microcline-KAlSi_3_O_8_, etc.) were recognized in most crusts. Gypsum was found in AS-G-VI, SM-G-VI, PA-G-VI, and E-G-VI (here in trace amounts). In addition, quartz was identified in AN-G-VI, A-G-VI, E-G-VI and PA-G-VI, albite in A-G-VI and PA-G-VI, microcline in AN-G-VI and E-G-VI, and muscovite (KAl_2_(AlSi_3_O_10_)_2_) in A-G-VI and E-G-VI. Traces of calcite were found in E-G-VI. Additionally, lead hydroxyapatite (Pb_5_(OH)(PO_4_)_3_) was detected in SM-G-VI and halite (NaCl) in A-G-VI.

**Table 3:** XRD composition of the BCs collected in historic buildings from Granada and Vigo

ID	Qz	Ab	Mc	Ms	Cal	Dol	Gp	Others
Granada’s BCs								
CAT-C-GR					+++		+++	
CC-T-GR					++++	+++	tr	
SJ-C-GR							++++	
SJD-MA-GR	++						++++	
SJP-T-GR							++++	
Vigo’s BCs								
AS-G-VI							++++	
SM-G-VI							++++	Lead hydroxyapatite (Pb_5_(OH)(PO_4_ )_3_) (+)
AN-G-VI	+++		++					
A-G-VI	+++	+++		tr.				Halite (NaCl) (tr)
E-G-VI	+++		+	+	tr		tr	
PA-G-VI	+++	++					++	

*Qz*, quartz (SiO_2_); *Ab*, albite (NaAlSi_3_O_8_); *Mc*, microcline (KAlSi_3_O_8_); *Ms*, muscovite (KAl_2_(AlSi_3_O_10_)(OH)_2_); *Cal*, calcite (CaCO_3_); *Dol*, dolomite (CaMg(CO_3_)_2_); *Gp*, gypsum (CaSO_4_·2H_2_O). + + + +: > 50%; + + +: 30–50%; + +: 10–30%; +: 3–10%; tr: < 3% (Check Table 1 to recognize the BCs IDs)

**Fig. 4:**
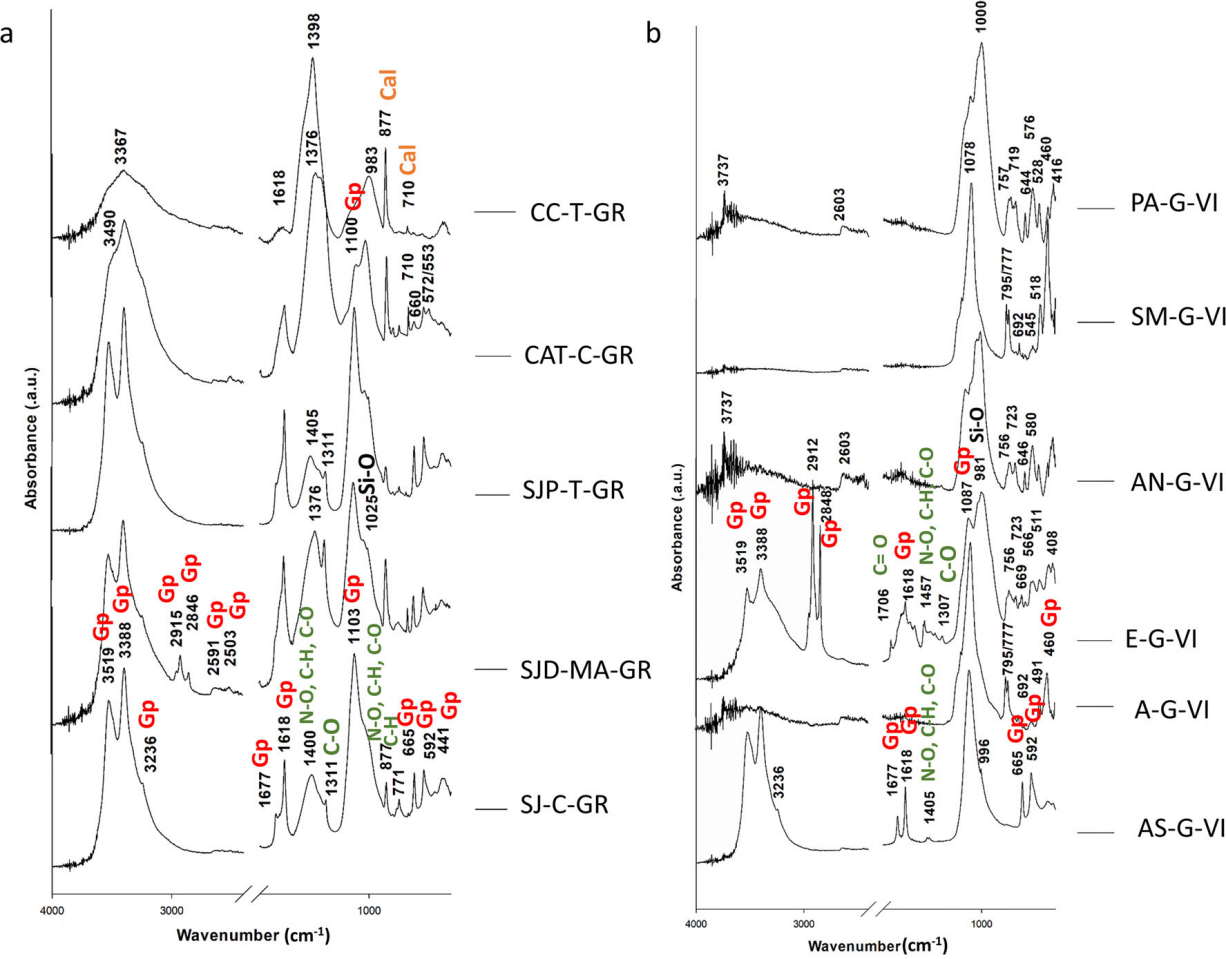
FTIR (absorbance) spectra of the BCs collected in historic buildings from Granada (**a**) and Vigo (**b**). *Gp*, gypsum; *Cal*, calcite (Check Table 1 to recognize the BCs IDs)

FTIR analysis confirmed most of the XRD results. Gypsum was identified through the absorption bands (Fig. 4) assigned to O-H functional groups (the doublet at 3519 and 3388 cm^−1^, a slight shoulder at 3236 cm^−1^, two weak bands at 2591 and 2503 cm^−1^, and the doublet at 1677 and 1618 cm^−1^) and S-O vibrations (the intense band at 1103 cm^−1^ and the weak bands at 665, 592, and 441 cm^−1^) (Socrates 2001; Lane 2007; Comite et al. 2020). Conversely to XRD, gypsum was found in A-G-VI and AN-G-VI using FTIR. This mismatching can be attributed to the gypsum concentration since XRD cannot identify minerals present at concentrations lower than 3% w. which is the technique threshold. However, contrariwise to that revealed by XRD, gypsum was not detected in PA-G-VI and SM-G-VI (Vigo’s BCs) using FTIR. This fact can be attributed to the compositional heterogeneity of these BCs samples and the absorption bands assigned to the forming minerals that can mask the bands associated to the gypsum.

Considering Granada’s BCs, calcite was identified in samples CC-T-GR, CAT-C-GR, SJD-MA-GR, and SJP-T-GR (this latter with weaker bands) due to the presence of simultaneous bands at 877 and 710 cm^−1^ (Ji et al. 2009) assigned to the asymmetric and symmetric CO_3_ deformation respectively. The other FTIR band assigned to calcite at 1400 cm^−1^ (asymmetric CO_3_ stretching vibration) cannot be used as representative feature due to the fact that ca. 1400–1370 cm^−1^, intense bands can be assigned to symmetric CH_3_ bending vibration which is indicative either of organic matter or to asymmetric NO_3_ stretching vibrations (Socrates 2001; Comite et al. 2020). The latter would suggest the presence of nitrate salts. The band at 771 cm^−1^ assigned to CH stretching (Socrates 2001) confirmed the presence of organic matter in all the Granada’s BCs except on CC-T-GR. Therefore, these bands may be representative of carbonaceous PM belonging to both microorganisms or soot from car engines. Conversely to XRD results, in the CC-T-GR sample, dolomite was not detected by FTIR, while an important amount of this mineral was found by XRD. FTIR bands used to identify dolomite are 3020 cm^−1^ and 2626 cm^−1^ as combination frequencies (Nguyen et al. 1991), and the band at 730 cm^−1^ assigned to the in-plane bending (*ν4*) mode of CO_3_^2−^ (Farmer 1974).

Regarding Vigo’s BCs, calcite was not identified by FTIR in E-G-VI as was done by XRD. This mismatching can be attributed to the overlapping of the calcite absorption bands with those attributed to silicate minerals. In the spectra of all Vigo’s BCs, with exception of the AS-G-VI sample, where only FTIR absorption bands assigned to gypsum were detected, bands associated to silicate were identified. These bands appeared mainly in the 1100–450 cm^−1^ (Socrates 2001; Rivas et al. 2012) with the following main features: a doublet of weak and strong bands in the region from 1100 to 950 cm^−1^, with the centroids of the bands registered at 1060 and 990 cm^−1^ respectively. In the FTIR spectra of A-G-VI and SM-G-VI samples, the Si–O symmetrical stretching vibrations observed at the doublet 795/777 cm^–1^, the Si–O symmetrical bending vibration at 692 cm^–1^ and the asymmetrical bending vibration observed at 460 cm^–1^, show the presence of quartz (Anbalagan et al. 2010). Moreover, the doublet at 795/777 cm^−1^ indicates that the silica is α-quartz (Marel and Bentelspacher 1976). This doublet is absent in PA-G-VI, AN-G-VI, and E-G-VI samples, where bands at 756 and 723 cm^−1^ were identified, also assigned to Si-O (Rivas et al. 2012). In the E-G-VI sample, a band at 1706 cm^−1^ corresponding to the stretching vibration C=O and at 1307 cm^−1^ corresponding to COC group vibration stretching, suggest the existence of carboxylic acids (Socrates 2001) and consequently, of organic matter. As was indicated for the Granada’s BCs, bands at 1400 cm^−1^ were identified in AS-G-VI and E-G-VI samples, which may be assigned to symmetric CH_3_ bending vibration or stretching vibration C=O (organic matter), as well as to asymmetric NO_3_ stretching vibrations (nitrate salts) (Socrates 2001, Comite et al. 2020).

**Fig. 5:**
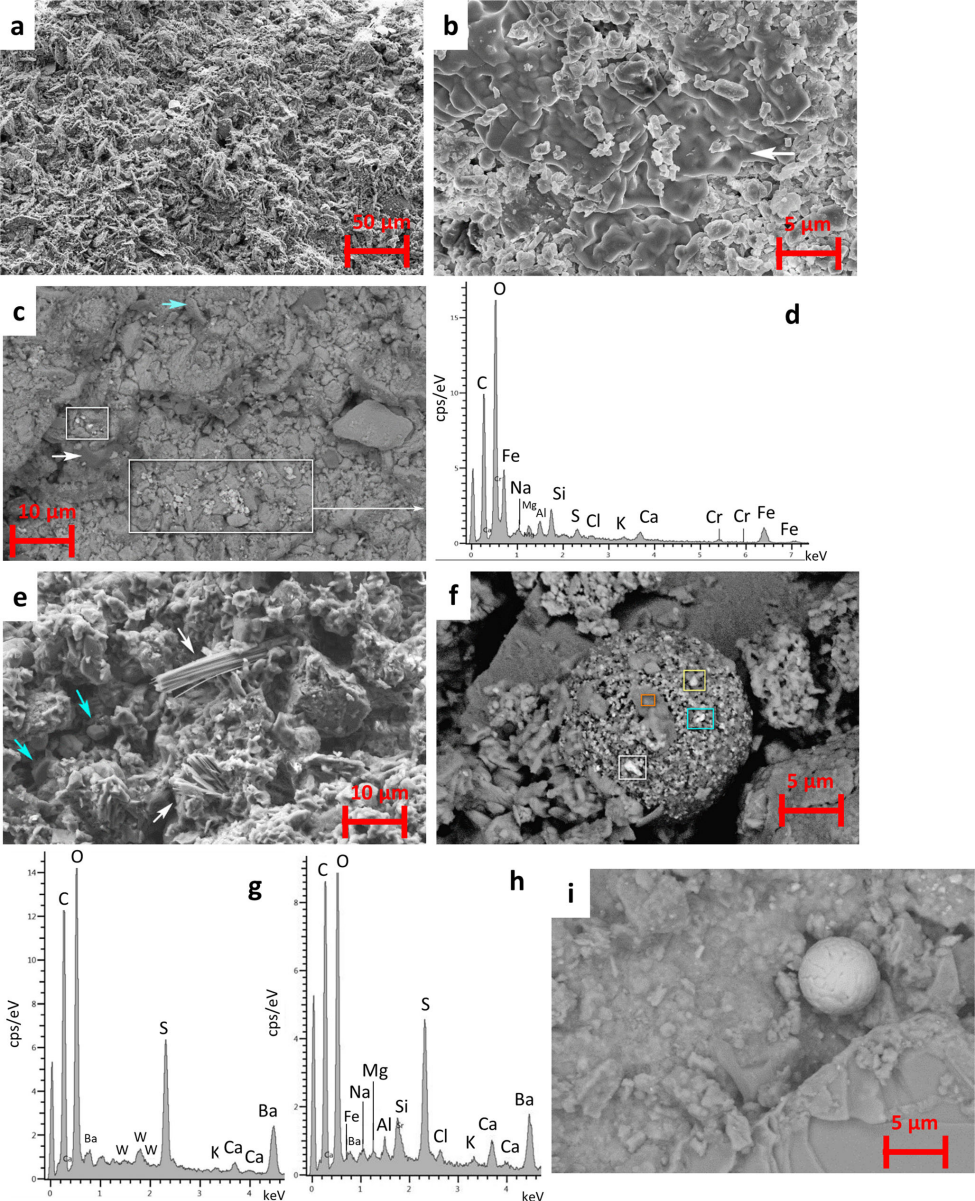
**a**–**d** HRSEM micrographs of calcarenite BCs from Granada. **a** SJ-C-GR showing the surface covered by interlocked gypsum plates (SE mode). **b** NaCl salt (white arrow) cementing rounded calcite crystals in the CAT-C-GR (BSE mode). **c** Detail from SJ-C-GR of clusters of particles made of Cr and Fe, NaNO_3_ salts (white arrow) and gypsum crystals (blue arrow). **d** EDX spectrum of particles inside the box of Fig. 4c. **e**–**h** HRSEM micrographs of the SJP-T-GR. **e** Lenticular and needle-shaped (white arrow) gypsum crystals and NaCl salts (blue arrow). **f** A composite carbonaceous rough sphere made of metal particles such as Fe (yellow box), gypsum (orange box), and soluble salts (NaCl). **g** EDX spectrum of white box shown in **f**, exhibiting Ca-sulfate and tungsten (W). **h** EDX spectrum of blue box shown in **f**, exhibiting Ca-, Ba-, and K-sulfates, NaCl, Sr, and Fe. **i** Smooth rounded Fe-rich particle from the CC-T-GR (For interpretation of the references to color in this figure legend, the reader is referred to the web version of this article)

The HRSEM-EDX analysis displayed that all BCs from Granada (Fig. 5) were made of an interlocking structure of arrowhead- and needle-like shapes of S and Ca endorsed to gypsum as was identified by XRD and FTIR, mixed primarily with quartz, clay minerals (aluminosilicates), and dolomite minerals. However, the study of the bulk BCs samples revealed the diverse micro-texture of the crusts according to the nature of their carbonate substrate, as well as the different chemical compositions of the embedded airborne particles, largely depending on the air quality scenario where the BCs were exposed in the city center.

The surface textural and chemical analysis performed by HRSEM-EDX in the rough BCs of the Monastery of *San Jerónimo* calcarenite-SJ-C-GR- showed abundant interlocked gypsum crystals with lenticular and plate-shaped habits (Fig. 5a), while on the BCs from the Cathedral-CAT-C-GR- agglomeration of rounded and disaggregated calcite grains of typically 2 μm in diameter or less were commonly found. EDX analyses revealed the existence of NaCl and NaNO_3_ salts in both types of crusts, which grew among the calcite grains and the gypsum crystals (Fig. 5b). Previous studies of airborne particles and weathered carbonate stones in the city of Granada show the occurrence of marine and secondary soluble salts as well as trace metals of diverse composition (Kontozova-Deutsch et al. 2011; Horemans et al. 2011; Urosevic et al. 2012; Potgieter-Vermaak et al. 2012). The chemical composition of those trace metals may varied from the one presented in here, considering that in this work, more diverse pollution scenarios were analyzed in the city center. Consequently, on CAT-C-GR, which was highly exposed to the impact of traffic, clusters of particles made either of Ca-phosphate and K-sulfate or composed of Ba-sulfate with Co and Sr were identified. Instead, in the BCs of the Monastery of *San Jerónimo*-SJ-C-GR- which were less exposed to the direct traffic effect, Ba-sulfate and Ca-phosphate were not found though Cr and Fe particles were detected (Fig. 5c, d).

The HRSEM-EDX study of the bulk travertine BCs revealed that the crust surface was mostly composed of lenticular and plate-shaped gypsum crystals about 2 μm in size, though clusters of needle-shaped gypsum crystals (ca. 30 μm in size) also developed (Fig. 5e). NaCl salts appeared all over the surface crust. Rough carbonaceous spheres made also of a mixture of diverse mineral phases (Fig. 5f), among them Ba-sulfate together with iron (Fe) and tungsten (W) particles (Fig. 5g, h) were found in the BCs most exposed to traffic, while only smooth Fe-rich particles were detected in BCs away from heavy traffic (Fig. 5i) (Urosevic et al. 2012). The strontium quarry *Montevive* located ca. 20 km from the city of Granada can be the natural source for W. Regarding the marble BC, the HRSEM-EDX analysis of the bulk BCs showed the presence of copious particles of diverse composition which often were found in clusters. Here, rounded particles rich in Ca-phosphate, K-sulfate, and Fe were identified, in addition to PbCl which was only found in the marble crusts. NaCl salts were abundant similarly to the BCs grown on calcarenite and travertine. Regarding their microstructure, while the BCs on marble commonly showed laminated structure, cauliflower-like BCs with globular morphology grown on travertine and calcarenite (Morillas et al. 2016b).

**Fig. 6:**
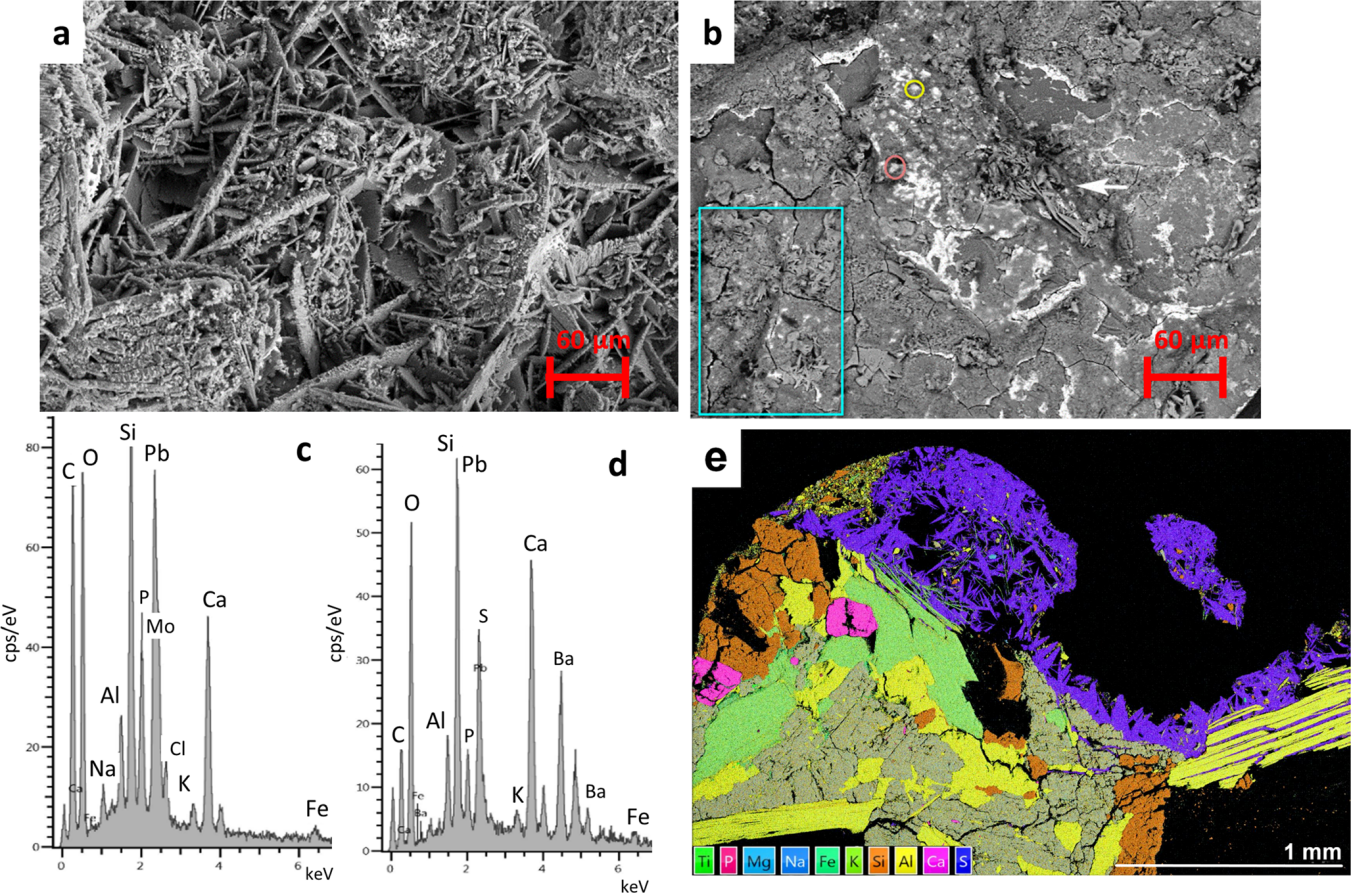
HRSEM micrographs of BCs from Vigo. **a** A rose-like formation of gypsum crystals in AS-G-GR crust. **b** AN-G-GR; note the fractured layer showing rose-like gypsum crystals (blue box) and larger lamellar gypsum crystals (white arrow) as well as copious metal particles (all bright spots in the image). **c** EDX analysis of orange circle in image **b**. Pb-Cl, Ca-phosphate, and Mo and Fe particles were detected. **d**: EDX analysis of yellow circle in image **b**. Ba-sulfate, K-sulfate, Ca-phosphate, as well as Cl and Fe particles were identified. **e** EDX false-color mineral map (thin section) of AS-G-VI; note the arrowhead gypsum crystals (purple color) which embeds scarce airborne metal particles (Pb and Ba) (For interpretation of the references to color in this figure legend, the reader is referred to the web version of this article)

The HRSEM-EDX study of the BCs from Vigo (Fig. 6) showed that the thick AS-G-VI crust was composed of rose-like masses of lenticular and plate-shaped gypsum crystals (Fig. 6a) that mostly embedded soot particles (as seen with PLM, Fig. 3h). NaCl was found in the BCS as in Granada, though in contrast very limited particles were only identified, namely Ba-sulfate, Ca-phosphate, and Pb-rich particles. Regarding the rest of the Vigo’s BCs, two types of surface textures were recognized applying HRSEM in the bulk samples. In addition to AS-G-VI, the A-G-VI crust taken from a building sited in a street close to the sea (N orientation) showed gypsum crystals matrix often displaying acicular or lenticular habits, occasionally arranged in rose-like clusters, which were less well-developed in A-G-VI crust. NaCl salt and framboidal Ca-phosfate rich particles were also identified in the A-G-VI crust along with Pb-Cl, Ba-sulfate, K-sulfate, and particularly Fe-rich particles. XRD also allowed the identification of halite (NaCl) in A-G-VI. In this crust, the metals detected with EDX were Co, Ti, Zn, Br, and Mo, though in very low amounts compared to the ones found in BCs located in streets with heavy traffic in the city center of Vigo.

The other surface textures observed by means of HRSEM-EDX in the bulk BCs from Vigo were those found in PA-G-VI, AN-G-VI, SM-G-VI, and E-G-VI crusts. Here, it was seen a non-continuous layer, made either of massive equidimensional tiny gypsum crystals or rose-like clusters, that was crossed by a polygonal crack network, and where the metal particles were particularly localized. Likewise, platy and bigger lamellar gypsum crystals grew among this fractured layer (Fig. 6b). These four BCs were the ones with more airborne metal particles, mainly the AN-G-VI crust. Though their chemical composition was quite alike, some differences were found in the PA-G-VI, AN-G-VI, SM-G-VI, and E-G-VI bulk crusts using EDX. The following particles were detected: Pb and Pb-Cl, Ba-sulfate, Ca-phosphate, and abundant Fe particles, together with the metals Zn and Ti, Co and W (PA-G-VI, E-G-VI crusts), and Mo (SM-G-VI, AN-G-VI crusts) (Fig. 6c, d). Anyhow, spherical metal particles were not observed in Vigo’s BCs unlike the Granada’s BCs. The detected heavy metal particles in this study can be derived from traffic-related materials (tire tread and brake dust), as well as from asphalt pavement (Si, Al, Ca, K) (Adachi and Tainosho 2004; Popoola et al. 2018; Comite et al. 2020).

The HRSEM-EDX analysis of the BCs prepared as thin sections allowed us to observe their cross section and by acquiring X-ray maps, the morphology, and distribution of their elements in depth. Fig. 6e displays the false-color mineral map of the thick AS-G-VI crust showing the lenticular and arrowhead gypsum crystals; Pb and Ba-sulfate rich particles were mainly located in the granite-crust interface.

The HRSEM-EDX analysis of the Granada’s BCs prepared as thin sections showed that crusts composition on the calcarenite, travertine, and marble was quite similar, largely made of S and Ca ascribed to gypsum which embedded copious minute Si particles credited for quartz grains (Figure S2a, b), and Fe-based particles which were spherical and plentiful on travertine crusts (Figure S2c). Also rounded metal particles mostly made of Si and Al were frequent. Minor amounts of aluminosilicates (Si/Al/Mg/K) endorsed to clay minerals were also present in all crusts as well as NaCl. False-color X-ray maps also revealed the abundance of Pb in the substrate-crust interface in the travertine (only in the CC-T-GR, close—though not directly exposed—to a heavy traffic street) where Pb was not necessarily related to Cl (Figure S2d). Likewise, Pb-rich and PbCl particles were identified on marble BCs unlike the calcarenite BCs.

**Fig. 7:**
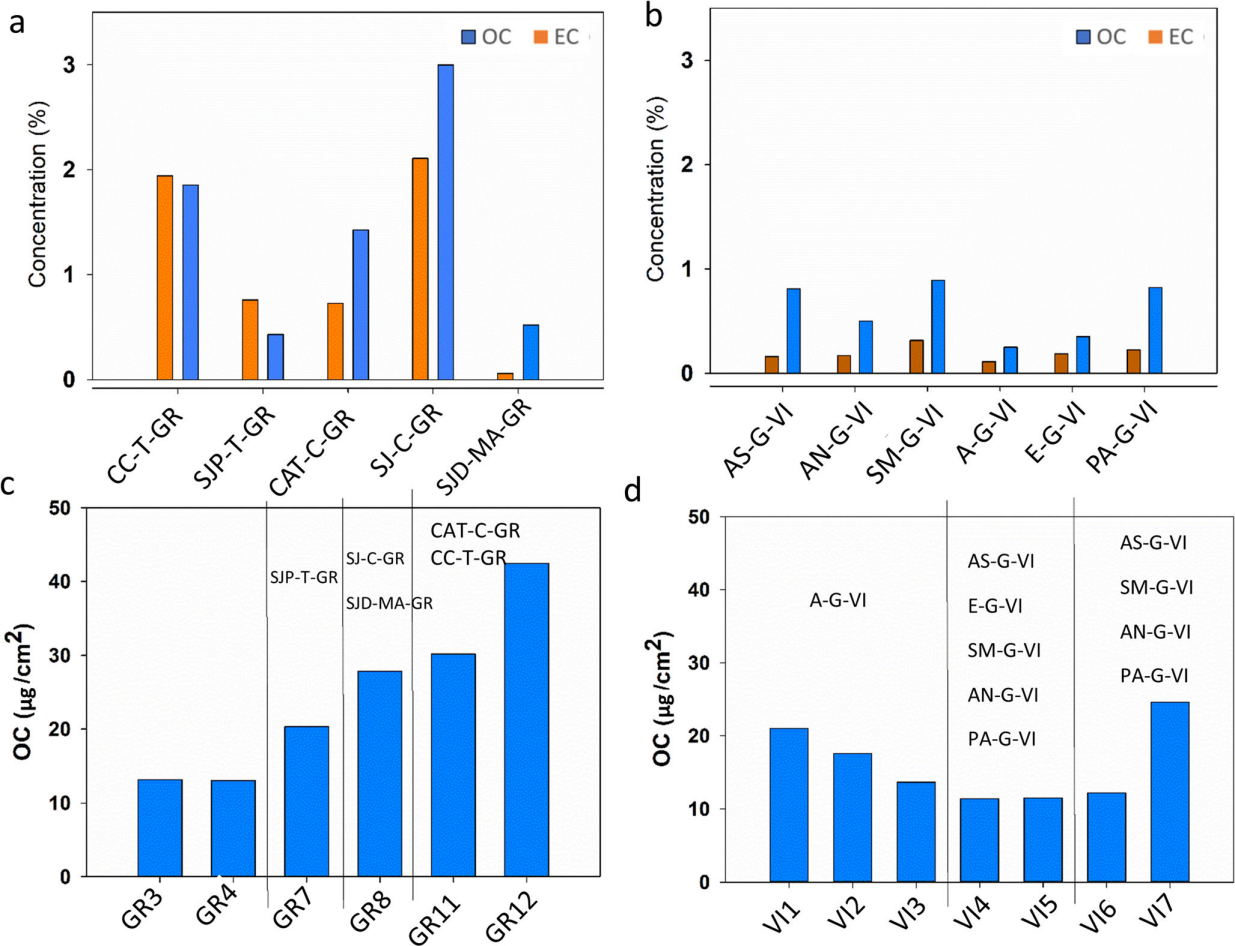
**a**, **b** Content of OC (organic carbon) and EC (elemental carbon) for the BCs collected in Granada and Vigo. **c**, **d** OC of the quartz fiber filters placed in both cities during 10 months. In the graphs corresponding to the filters (**c**, **d**), the BCs collected in the surroundings where the filters were placed are shown. Check Tables 1 and 2 to recognize the BCs- and filters- IDs (For interpretation of the references to color in this figure legend, the reader is referred to the web version of this article)

As regards the carbonaceous components, they were more abundant in Granada’s BCs than these in Vigo’s BCs (Fig. 7a, b). In fact, OC represented 1.40 wt% and 0.50 wt% in Granada and Vigo samples, respectively, while EC represented 1.12 wt% and 0.18 wt% respectively (Granada also contained higher PM concentrations- Figure S1). This finding agrees with the results acquired with stereomicroscopy, PLM, FTIR, and HRSEM-EDX, because more soot particles were found in Granada’s BCs than in Vigo’s BCs. It is well known from the literature that soot particles are composed by EC (Piazzalunga et al. 2013; Fermo et al. 2015). The lower carbonaceous content of Vigo’s BCs compare to Granada’s BCs can be explained by the small atmospheric PM concentrations of the city. Although Vigo is an industrial city, its sea-exposed situation promotes lower pollution levels.

The confirmation that the BCs composition was linked to the different environmental conditions of these two cities is given by the OC/EC ratio, which is 0.6–2 for Vigo while varies between 1.25 and 3.7 in Granada (Saarikoski et al. 2008; Kanakidou et al. 2010; Cheng et al. 2016). Higher values of OC/EC ratio are assigned to the formation of secondary organic species (Daellenbach et al. 2016).

In order to study the PM deposition on the stone substrates, quartz fiber filters are commonly used as surrogates (Fermo et al. 2018). Comparing the chemical composition of the atmospheric PM collected from the filters (Fig. 7c, d) with that collected from BCs (Fig. 7a, b), it is worth noting that EC was not detected in the PM from the filters. This fact could be assigned to the sampling height: while BCs were taken from the bottom parts of the buildings, filters were placed at higher heights. Since EC is mainly due to traffic (Fuzzi et al. 2006; Gentner et al. 2012; Piazzalunga et al. 2013), the location of the filters could explain why EC was not deposited on them; indeed, filters were placed higher than the areas from which BCs were taken. Another hypothesis is that EC, conversely to OC, is less retained on the filter surfaces, probably due to the different hydrophobicity of the carbonaceous compounds. Indeed, in a case study conducted by Fermo et al., (2018) in Milan (Italy), this fact was also observed when comparing stone surfaces with quartz fiber filters exposed under the same conditions.

**Fig. 8:**
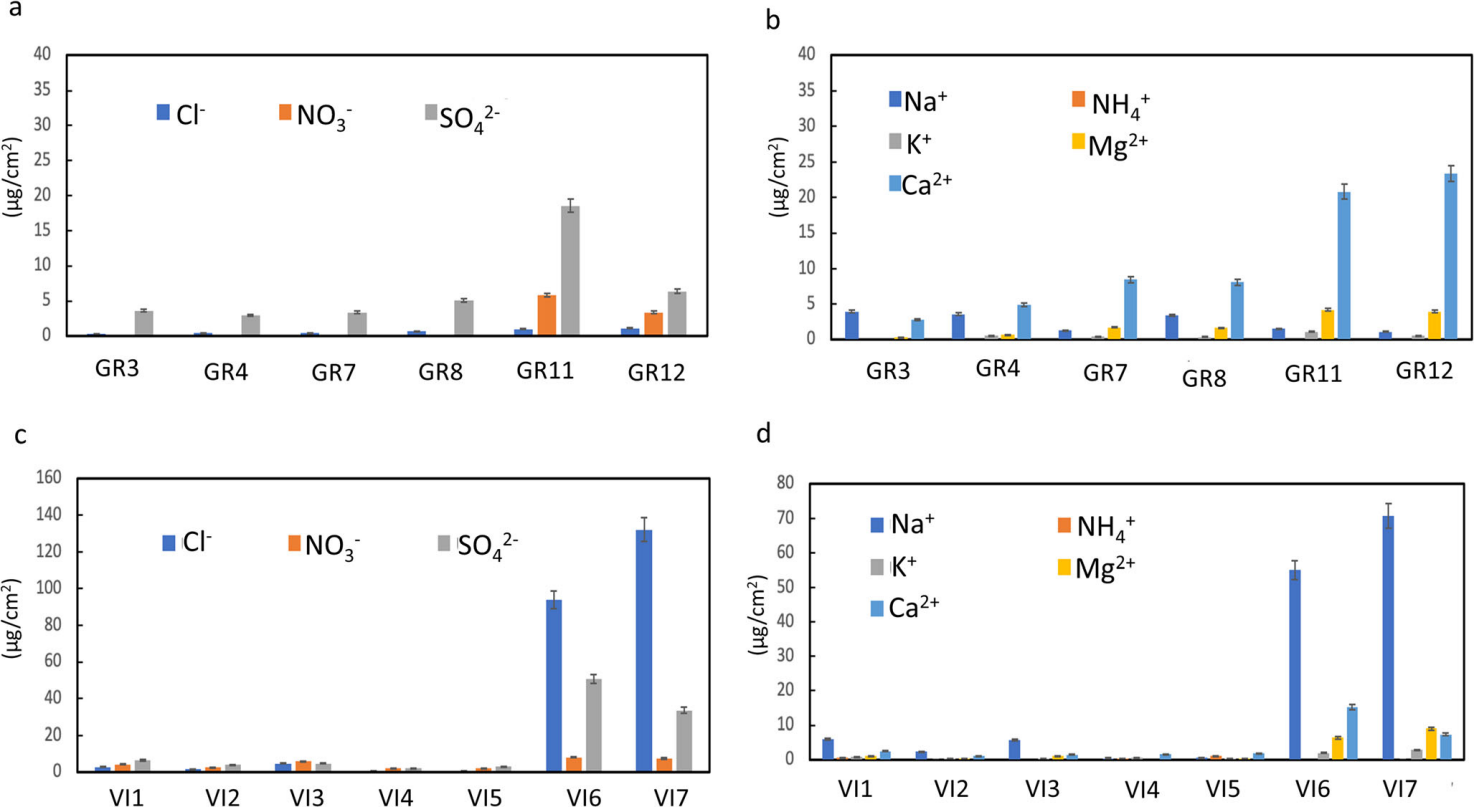
Content of main anions and cations (Cl^−^, SO_4_^2−^, NO_3_^−^, Na^+^, NH_4_^+^, K^+^, Mg^+^, and Ca^2+^) on quartz fiber filters collected in Granada (**a**, **b**) and Vigo (**c**, **d**) (Check Table 2 to recognize the filters IDs. For interpretation of the references to color in this figure legend, the reader is referred to the web version of this article)

**Fig. 9:**
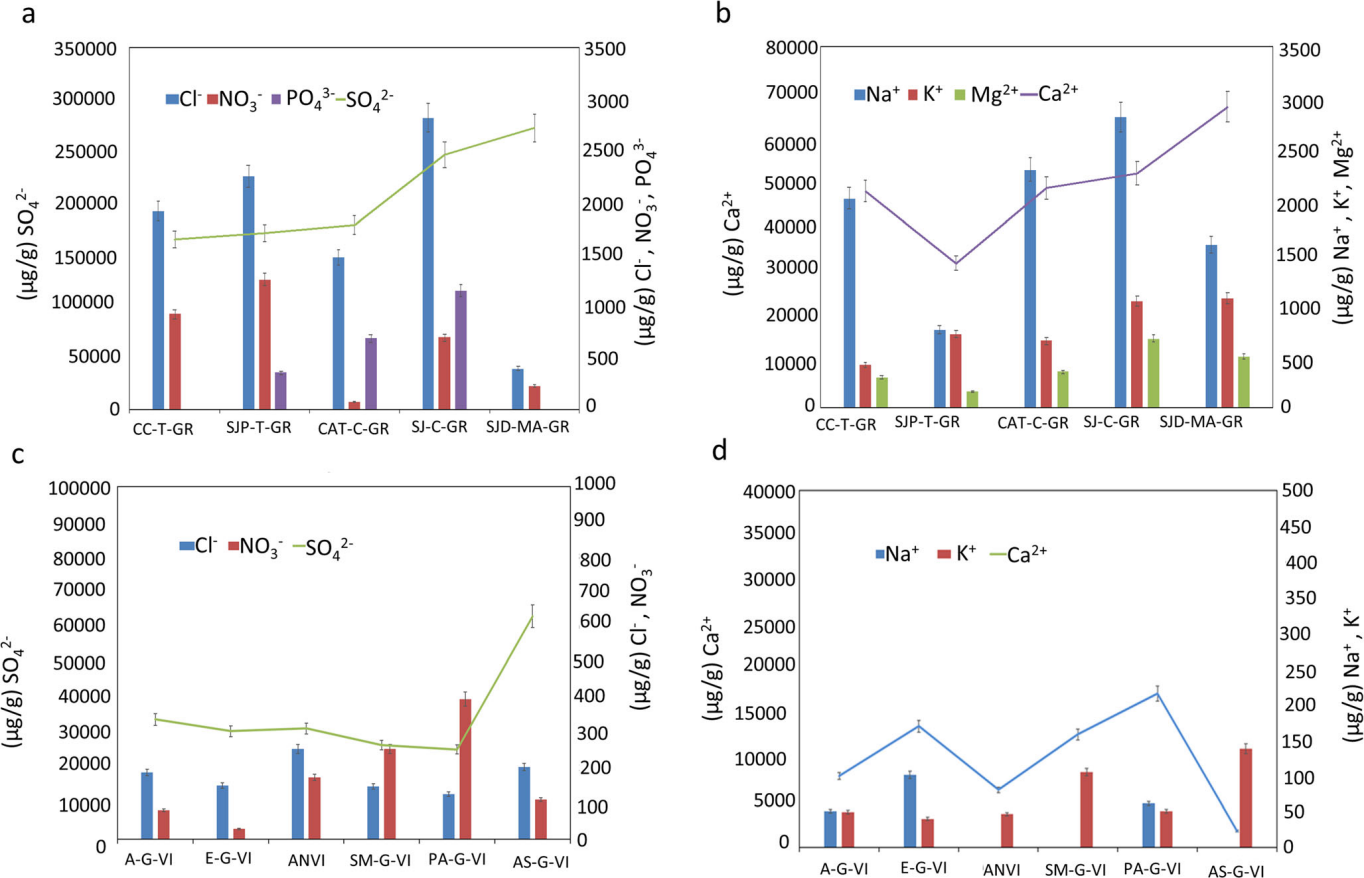
Content of main anions and cations (Cl^−^, NO_3_^−^, SO_4_^2−^, PO_4_^3−^, Na^+^, K^+^, Mg^2+^, and Ca^2+^) on BCs collected in Granada (**a**, **b**) and Vigo (**c**, **d**) (Check Table 1 to recognize the BCs IDs. For interpretation of the references to color in this figure legend, the reader is referred to the web version of this article)

Considering that an evident relationship between filters and BCs was observable as regards the carbonaceous components content, the type of substrate and the specific exposure conditions seem to be involved in the BCs genesis. Likewise, a comparison between filters and BCs was made considering the main ions (Cl^−^, SO_4_^2−^, NO_3_^−^, PO_4_^3−^, Na^+^, NH_4_^+^, K^+^, Mg^+^, and Ca^2+^) present in the PM collected from both the quartz filters (Fig. 8) and the BCs (Fig. 9).

In Granada’s filters, Ca^2+^ and SO_4_^2−^ (Fig. 8a, b) were the ions present in higher concentrations in accordance to reported results in previous studies for other sites where these two species were the main contributors (Urosevic et al. 2012; Fermo et al. 2015, 2018). Despite Granada is located ca. 50 km from the coast, a marine influence was found due to the Cl^−^ detected in the PM collected from filters (Fig. 8a), in agreement with Urosevic et al. (2012). Na^+^ and Mg^2+^ were also detected in the PM (Fig. 8b). In GR11 and GR12, NO_3_^−^ was identified (Fig. 8a) contrary to the other filters from the same city. Likewise, according to the observations found in a previous case study on filters exposed for months to atmospheric pollution (Fermo et al. 2018), the NH_4_^+^ present in the current research could come from ammonium sulfate ((NH_4_)_2_SO_4_) and ammonium nitrate (NH_4_NO_3_). Hence, as already observed in Fermo et al. (2018), the decomposition of (NH_4_)_2_SO_4_ occurred on the filters and the same happened in the BCs, bringing to the formation of some acidity due to the salt hydrolysis. In Granada’s BCs, SO_4_^2−^ is the main ion with notably lower concentrations of Cl^−^ and NO_3_^−^ (Fig. 9a, b). Likewise, PO_4_^3−^ was found in SJP-T-GR, CAT-C-GR, and SJ-C-GR in accordance with the results obtained by HRSEM-EDX analysis.

In the PM collected from BCs in Vigo (Fig. 8c, d) Cl^−^ was the ion present in the highest concentration followed by Na^+^ and SO_4_^2−^. This composition is not surprising taking into account that Vigo receives the direct influence of marine aerosol; consequently, the presence of NaCl was expected. Nevertheless, it is worth noting that for samples AN-G-VI, SM-G-VI, and AS-G-VI, Na^+^ was not detected (Fig. 9c, d).

In general, what has been observed comparing filters and BCs is that salts transformation occurred on the stone surfaces. Although high Cl^−^ concentrations were detected in Vigo’s filters, due to the marine aerosol, BCs from Vigo showed in general a significantly low Cl^−^ concentration because a dissolution phenomenon happened due to the washout related to the intense rain episodes in this region.

A further interesting observation that can be drawn from the comparison of BCs compositions in these two cities is that while in Granada there was a quite good correlation between the Ca^2+^ and SO_4_^2−^ trends, this fact was not detected in Vigo where SO_4_^2−^ seemed to show a trend more similar to that of K^+^. As was reported in Matović et al. (2012), syngenite (K_2_Ca(SO_4_)_2_·H_2_O) is a common secondary deposit on black crust developed in granite monuments. However, K_2_Ca(SO_4_)_2_·H_2_O was not detected by XRD in the BCs collected in Vigo, most likely because its concentration was below the technique detection limit. All in all, the presence of salts on BCs is harmful since these hygroscopic compounds induce further degradation phenomenon.

## Conclusions

The composition and microstructure of BCs collected in Vigo and Granada (Spain) on substrates of different nature, i.e., granite, limestone, travertine, and marble, depend on the availability of Ca^2+^ from the underneath stone, the accumulation time of atmospheric pollutants on their surfaces, and the diverse air quality scenarios they are exposed. The predominant dry environment of Granada leads to the development of thick BCs on carbonate stones while in Vigo, the intense and common rain episodes preclude the formation of thick BCs on the granitic walls. Consequently, BCs from Granada show more complex structure and darkness, particularly those sampled from substrates where the impact of traffic pollution has been more intense. On the other hand, though Granada city is ca. 50 km from the Mediterranean coast, sea-salt aerosols have been widely found embedded in the BCs, such as NaCl and secondary NaNO_3_ salts. The latter were not found in Vigo’s BCs. Additionally, in all the studied BCs from both cities, Pb-Cl and Ca-Cl-rich particles, Ca-phosphate particles, and clusters of Ba-sulfate-rich particles were detected. Moreover, metal-rich rounded particles were more abundant in Granada’s BCs, including soot particles. BCs from Granada were richer in carbonaceous components (OC and EC) than those from Vigo. Although in the filters PM did not show EC-related to traffic, in the BCs from both cities, OC and EC were detected. The discrepancy arises because of the different heights of the BCs sampling (at the bottom of the façades) and the higher location of the filters.

The different compositions detected in the BCs should be ultimately related with the dissimilar pollution scenarios of the studied cities. Higher air pollution characterizes the non-industrialized Granada which is favored by its topography and semi-continental climate, in contrast to the industrial and Atlantic Ocean-exposed city of Vigo.

Moreover, the polymineral composition of the Vigo’s granite hinders the formation process of gypsum crystals comparing to the easiest development of gypsum crystals in the carbonate stones used in Granada historical buildings.

## Supplementary information

ESM 1 (DOCX 3378 kb)

## Data Availability

All data generated or analyzed during this study are included in this published article.
