# Supplementary material for: Characterization of black crusts developed on historic stones with diverse mineralogy under different air quality environments
Source: Environ Sci Pollut Res Int. 2021 Jul 24;29(20):29438–54. doi: 10.1007/s11356-021-15514-w (PMC9001199; doi:10.1007/s11356-021-15514-w)
Supplement: Supplementary file 1 — (DOCX 3378 kb) [file 11356_2021_15514_MOESM1_ESM.docx]

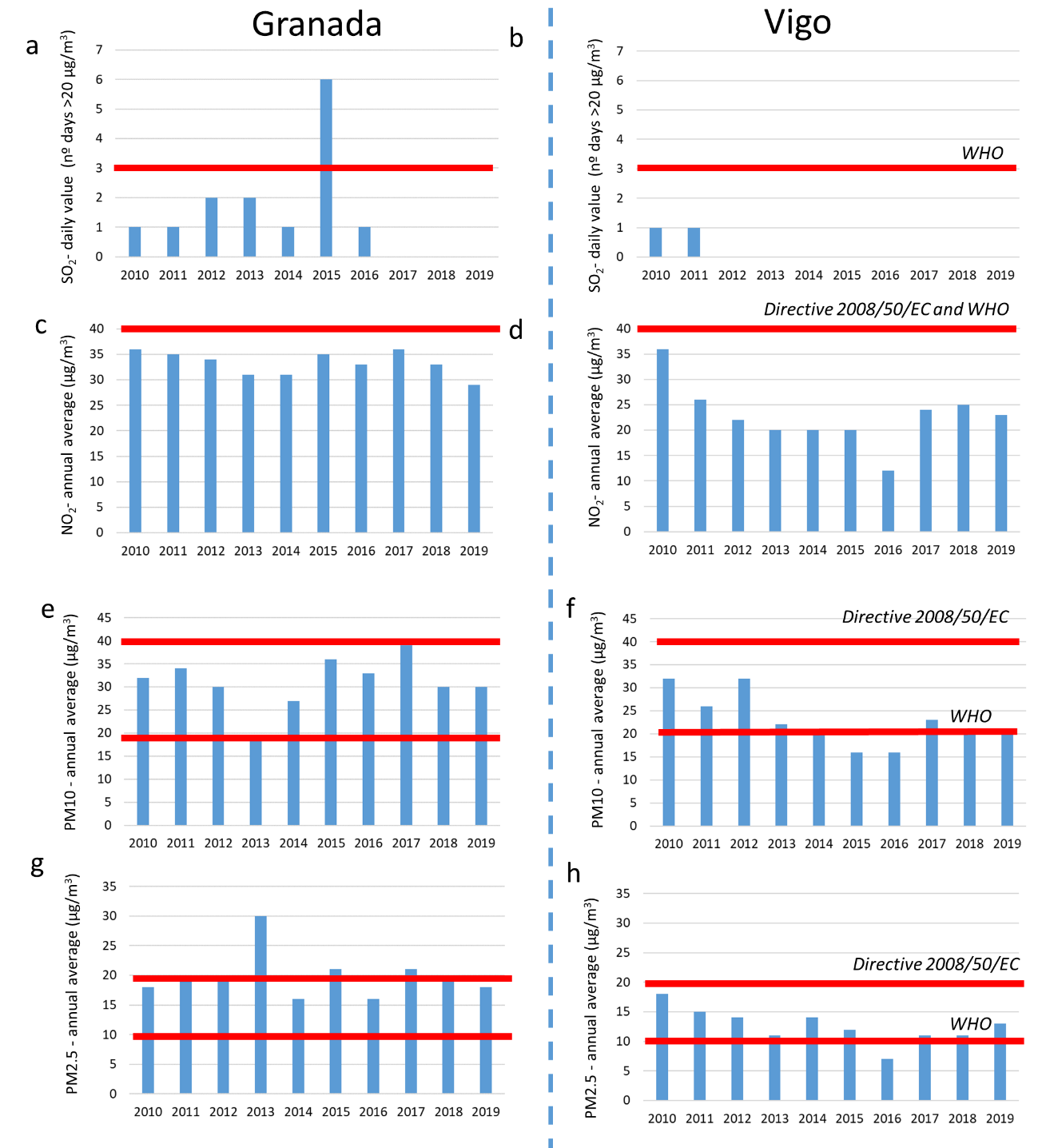


Figure S1: Air pollution values (SO_2_, NO_2_ and PM10 and PM2.5) from 2010-2019 in the city center of Granada and Vigo (Spain). Threshold values set by the WHO (World Health Organization) which are more restrictive than those established by the Directive 2008/50/EC, are shown. Data from *Informes the Ecologistas en Acción* 2010-2019.


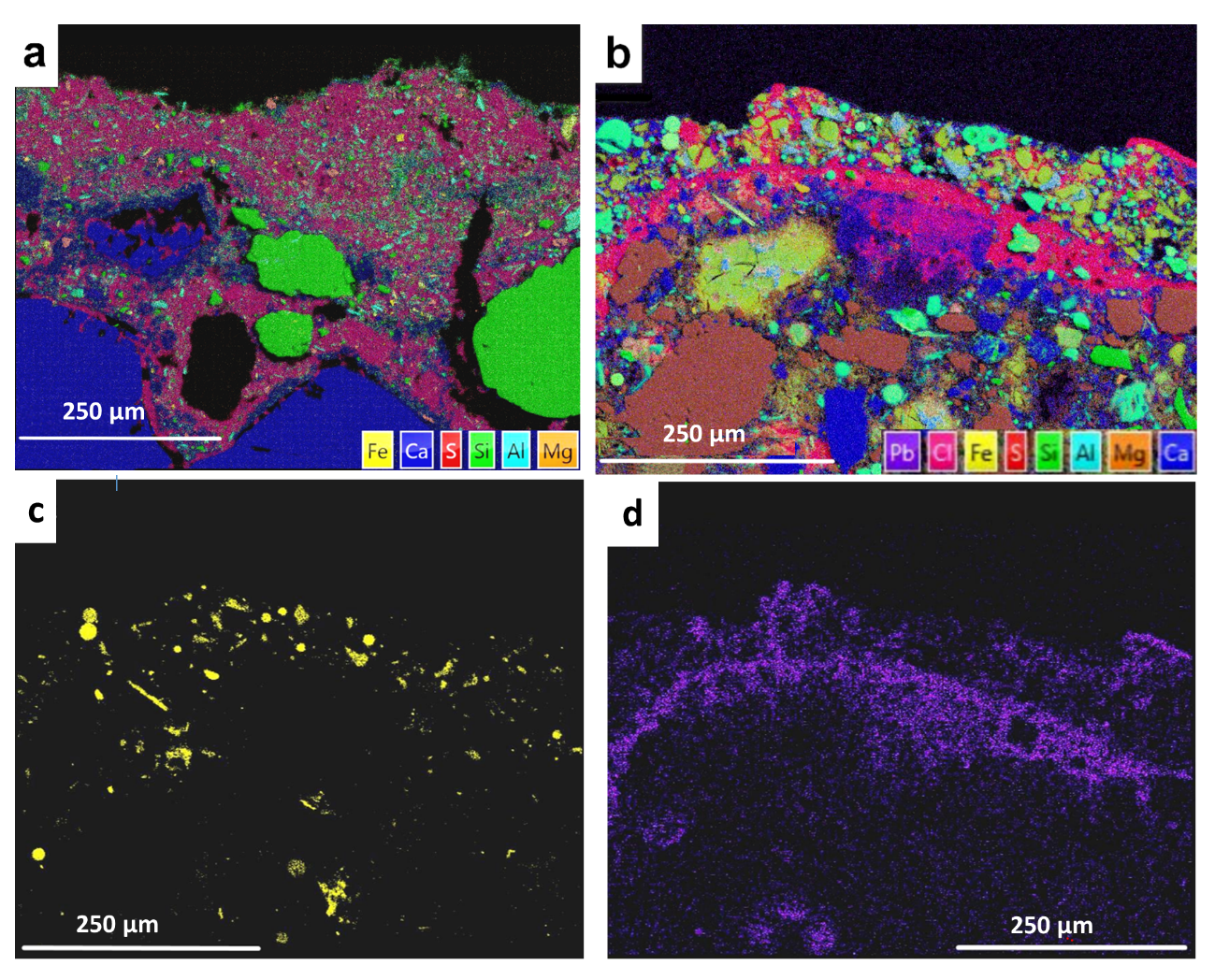


Figure S2. HRSEM-EDX false-color elemental maps of BCs on carbonate stones. a: SJD-MA-GR; note the gypsum-rich matrix embedding quartz and clay minerals grains, and Fe-rich particles. b-d) CC-T-GR. b: X-ray map showing the distribution, morphology and composition of particles trapped into the gypsum matrix; note the many Si- and Al-rich spherical particles. c: X-ray map of Fe showing its presence in the rounded particles. d: X-ray map of Pb exhibiting its location in the travertine-crust interface (For interpretation of the references to color in this figure legend, the reader is referred to the web version of this article).
